# Fermentation properties and potential prebiotic activity of Bimuno^®^ galacto-oligosaccharide (65 % galacto-oligosaccharide content) on *in vitro* gut microbiota parameters

**DOI:** 10.1017/S0007114516002269

**Published:** 2016-06-08

**Authors:** Roberta Grimaldi, Jonathan R. Swann, Jelena Vulevic, Glenn R. Gibson, Adele Costabile

**Affiliations:** 1 Department of Food and Nutritional Sciences, University of Reading, Reading RG6 6AP, UK; 2 Division of Computational and Systems Medicine, Imperial College, London SW7 2AZ, UK; 3 Clasado Research Services Ltd, Science & Technology Centre, University of Reading, Reading RG6 6BZ, UK; 4 Life Sciences Department, Health Sciences Research Centre, Whitelands College, University of Roehampton, London SW15 4JD, UK

**Keywords:** Prebiotic activity, Bimuno^®^ galacto-oligosaccharides, Microbiota composition, Fluorescence *in situ* hybridisation, SCFA

## Abstract

Prebiotic oligosaccharides have the ability to generate important changes in the gut microbiota composition that may confer health benefits to the host. Reducing the impurities in prebiotic mixtures could expand their applications in food industries and improve their selectivity and prebiotic effect on the potential beneficial bacteria such as bifidobacteria and lactobacilli. This study aimed to determine the *in vitro* potential fermentation properties of a 65 % galacto-oligosaccharide (GOS) content Bimuno^®^ GOS (B-GOS) on gut microbiota composition and their metabolites. Fermentation of 65 % B-GOS was compared with 52 % B-GOS in pH- and volume-controlled dose–response anaerobic batch culture experiments. In total, three different doses (1, 0·5 and 0·33 g equivalent to 0·1, 0·05 and 0·033 g/l) were tested. Changes in the gut microbiota during a time course were identified by fluorescence *in situ* hybridisation, whereas small molecular weight metabolomics profiles and SCFA were determined by ^1^H-NMR analysis and GC, respectively. The 65 % B-GOS showed positive modulation of the microbiota composition during the first 8 h of fermentation with all doses. Administration of the specific doses of B-GOS induced a significant increase in acetate as the major SCFA synthesised compared with propionate and butyrate concentrations, but there were no significant differences between substrates. The 65 % B-GOS in syrup format seems to have, in all the analysis, an efficient prebiotic effect. However, the applicability of such changes remains to be shown in an *in vivo* trial.

*In vitro* and *in vivo* studies involving prebiotic oligosaccharides have been carried out using inulin and its fructo-oligosaccharide (FOS) derivatives, as well as various galacto-oligosaccharides (GOS). It has been shown that these food ingredients have the ability to improve selectively the growth of bifidobacteria, and consequently lead to important changes in the gut microbiota composition that may confer health benefits to the host. To date, GOS has been associated with numerous health benefits such as low energy content, insulin-independent metabolism and stimulation of growth and metabolism of specific colonic microbiota. The two main mechanisms by which this is achieved are through the production of SCFA from its fermentation and the selective enhancement of beneficial gut organisms^(^ ^1^ ^)^. GOS can be defined as a mixture of the end products of lactose breakdown by *β*-galactosidases, containing two to eight saccharide units, with a terminal glucose unit^(^ ^2^ ^)^. These mixtures can be complex and their structures are often imperfectly characterised. They tend to be mixtures of *β*-1,3, *β*-1,4 and *β*-1,6 linkages with degrees of polymerisation ranging from two to five. A characteristic of GOS is that the set of structures present depends on the source of the enzyme used to bring about synthesis. The structural and functional relationship of GOS plays a role in targeting the *Bifidobacterium* genus^(^ ^3^ ^)^. Another important aspect is the presence of impurities such as monosaccharaides, disaccharides or metabolic products from purification steps. Removing these compounds can lead to a mixture with a GOS content as high as possible that can be better used to study fermentation and structural properties of novel prebiotics in *in vitro* experiments. In addition, purified Bimuno^®^ GOS (B-GOS) mixture might have significantly increased interest in production and application in various food and pharmaceutical processes, especially if the prebiotic is incorporated directly into food such as diabetic or low-energy foods. Reducing monosaccharides and disaccharides such as glucose, galactose and lactose from the mixture might help minimise their impact on consumers, particularly by taking into account lactose intolerance. Several techniques have been suggested in order to obtain high recovery of GOS, but all of them have some limitations^(^ ^4^ ^)^. B-GOS (Bimuno^®^ 52 % GOS content; Clasado Biosciences Ltd) refers to prebiotic GOS compounds that have multiple biological health activities within the colonic environment. It is produced from the activity of galactosidase enzymes isolated from *Bifidobacterium bifidum* NCIMB 41171^(^ ^5^ ^)^. The method for B-GOS purification has been studied by Goulas *et al.* ^(^ ^6^ ^)^ in order to reduce the amount of free glucose and galactose produced during its synthesis, and it led to the removal of 92 % of glucose by fermentation with *Saccharomyces cerevisiae*. The bifidogenic properties of B-GOS have been investigated *in vitro* and *in vivo*. Tzortzis *et al.* ^(^ ^7^ ^)^ showed *in vitro* and in a pig trial the prebiotic potential of GOS. In this study, it was established that B-GOS prebiotic activity was relevant in terms of increase in bifidobacteria numbers, SCFA production and decreased pH, compared with other prebiotics such as inulin and other GOS types^(^ ^7^ ^)^. B-GOS has also been tested in healthy volunteers, clinical conditions that have a purported microbial aetiology such as irritable bowel syndrome, traveller’s diarrhoea and obesity, and on cognitive functions^(^ ^8^ ^–^ ^13^ ^)^. This study aimed to determine the potential prebiotic activity of a purified 65 % GOS content B-GOS, compared with 52 % GOS content B-GOS, used as positive control in *in vitro* dose–response batch cultures.

## Methods

### Substrates

The two B-GOS products, supplied by Clasado BioSciences Ltd, used in this study were produced from the activity of galactosyltransferases from *B. bifidum* NCIMB 41171 on lactose^(^ ^14^ ^)^. Both were used in syrup form. The B-GOS mixtures consisted of (w/w) 52 % GOS, 8 % lactose, 22 % glucose, 16·5 % galactose and 65 % GOS, 10·1 % lactose, 22 % glucose, 1·8 % galactose, respectively.

### Faecal inoculation

Experiments were carried out using fresh faecal samples from three healthy donors (one female aged 26 years and two males aged 25 and 31 years, respectively) who were free of any metabolic and gastrointestinal diseases, were not taking probiotic or prebiotic supplements and had not taken antibiotics for 6 months before faecal sample donation. All donors then provided written informed consent, and filled in a standard questionnaire to collect information regarding health status, drug use, clinical anamnesis and lifestyle factors. This study was approved by The University of Reading Research Ethics Committee (UREC 15/20). Faecal samples were placed in an anaerobic jar (AnaeroJar™ 2.5L; Oxoid Ltd) including a gas-generating kit (AnaeroGen™; Oxoid). Samples were diluted 1/10 w/w in anaerobic PBS (0·1 mol/l phosphate buffer solution, pH 7·4) and homogenised (Stomacher 400; Seward) for 2 min at 240 paddle beats/min. Samples were added to anaerobic fermenters within 15 min of voiding.

### *In vitro* batch culture fermentation

This method was previously described by Rycroft *et al.* ^(^ ^15^ ^)^. B-GOS were added at concentrations shown in Table 1 before adding 5 ml of faecal slurry (final concentration of 1 %). An extra vessel with no added carbohydrate source was also included as a negative control. The pH was maintained in the range of 6·7–6·9 via pH controllers (Fermac 260; Electrolab) and automatically adjusted by adding 0·5 mm-NaOH and HCl to the vessels when required. The pH and temperature mimicked the conditions of the distal region of the human large intestine. Batch culture fermentations were run for 24 h, and the samples (3·5 ml from each vessel) were collected at 0, 4, 8 and 24 h for analysis of bacterial populations and metabolite production. Fermentation experiments were performed in triplicate.

**Table 1:** Doses of the Bimuno^®^ galacto-oligosaccharide (B-GOS) syrups tested (equivalent to 0·1, 0·05 and 0·033 g/l) in 100 ml working volume vessels during 24 h of fermentation in pH- and volume-controlled batch fermentation experiments

Vessel	Treatment	Syrup (g)	DM	GOS in 100 ml
1	B-GOS (52 % GOS content)	2·66	2	1
2	B-GOS (52 % GOS content)	1·33	1	0·5
3	B-GOS (52 % GOS content)	0·88	0·66	0·33
4	B-GOS (65 % GOS content)	2·05	1·54	1
5	B-GOS (65 % GOS content)	1·02	0·77	0·5
6	B-GOS (65 % GOS content)	0·68	0·51	0·33
7	Negative control (only faeces)			

### Bacterial enumerations by fluorescence *in situ* hybridisation

Differences in bacterial populations were assessed by fluorescence *in situ* hybridisation (FISH) with oligonucleotide probes designed to target specific diagnostic regions of 16S rRNA, as previously described^(^ ^16^ ^)^. The probes were commercially synthesised and labelled at the 5′ end with the fluorescent dye Cy3 (Sigma-Aldrich) as reported in Table 2 ^(^ ^17^ ^–^ ^22^ ^)^. Numbers of specific and total bacteria were determined using the following equation: DF×ACC×6732·42×50×DF sample, where DF is the dilution factor (300/375=0·8), ACC is the average cell count of fifteen fields of view and DF sample refers to the dilution of sample used with a particular probe or stain. The figure 6732·42 refers to the area of the well divided by the area of the field of view and the factor 50 takes the cell count back to per millilitre of sample.

**Table 2:** Oligonucleotide probes used in the study for fluorescence *in situ* hybridisation analysis of bacterial populations

Probe name and sequence (5′–3′)	Target species	Hybridisation-washing temperature (°C)	References
Bif164 CATCCGGCATTACCACCC	Most *Bifidobacterium* spp. and *Parascardovia denticolens*	50–50	^(^ ^20^ ^)^
Bac303 CCAATGTGGGGGACCTT	Most *Bacteroides sensu stricto* and *Prevotella* spp.; all *Parabacteroides*; *Barnesiella viscericola* and *Odoribacter splanchnicus*	46–48	^(^ ^21^ ^)^
Lab158 GGTATTAGCAYCTGTTTCCA	Most *Lactobacillus*, *Leuconostoc* and *Weissella* spp.; *Lactococcus lactis*; all *Vagococcus*, *Enterococcus*, *Melissococcus*, *Tetragenococcus*, *Catellicoccus*, *Pediococcus* and *Paralactobacillus* spp.	50–50	^(^ ^22^ ^)^
Chris150 TTATGCGGTATTAATCTYCCTTT	Most members of *Clostridium* cluster I; all members of *Clostridium* cluster II; *Clostridium tyrobutyricum*; *Adhaeribacter aquaticus* and *Flexibacter canadensis* (family *Flexibacteriaceae*); (Eubacterium) *combesii* (family *Propionibacteriaceae*)	50–50	^(^ ^23^ ^)^
Erec 482 GCTTCTTAGTCARGTACCG	Most members of *Clostridium* cluster XIVa; *Syntrophococcus sucromutans,* (Bacteroides) *galacturonicus* and (Bacteroides) *xylanolyticus*, *Lachnospira pectinschiza* and *Clostridium saccharolyticum*	50–50	^(^ ^23^ ^)^
EUB338 I GCTGCCTCCCGTAGGAGT	Total bacteria	46–48	^(^ ^24^ ^)^
EUB338 II GCAGCCACCCGTAGGTGT	Total bacteria	46–48	^(^ ^24^ ^)^
EUB338 III GCTGCCACCCGTAGGTGT	Total bacteria	46–48	^(^ ^24^ ^)^
Rrec584 TCAGACTTGCCGYACCGC	*Roseburia – Eubacterium rectale* (a component of cluster XIVa)	50–50	^(^ ^25^ ^)^
Prop853 ATTGCGTTAACTCCGGCAC	*Clostridium* cluster IX	50–50	^(^ ^25^ ^)^

### SCFA analysis

Production of SCFA was determinate using GC as previously described^(^ ^23^ ^)^. Peaks were integrated using Agilent ChemStation software (Agilent Technologies), and SCFA content was quantified by single-point internal standard method. Peak identity and internal response factors were determined using a 20-mm calibration cocktail including acetic, propionic, iso-butyric, butyric, iso-valeric, valeric, caproic and caprylic acids.

### Metabolite analysis by ^1^H-NMR

Fermentation supernatants from all time points were defrosted, vortexed and centrifuged at 599 ***g*** for 5 min. Supernatants were filtered using 0·22-µm low protein binding durapore polyvinylidene fluoride membrane (Millex; EMD Millipore) and 400 μl was transferred into fresh eppendorf tubes. Filtered samples were then combined with 200 μl of phosphate buffer (0·2 m (pH 7·4) in D_2_O plus 0·001 % trimethylsilyl propionate (TSP)). The mixture was vortexed and centrifuged at 1136 ***g*** for 10 min and then 550 μl was transferred into 5-mm NMR tubes for analysis. All NMR spectra were acquired on Bruker Avance DRX 500 MHz NMR Spectrometer (Bruker BioSpin) operating at 500 MHz. They were acquired using a standard 1-dimensional (1D) pulse sequence (recycle delay (RD)−90°−t1−90°−tm−90°−acquire free induction decay (FID)) with water suppression applied during RD of 2 s, a mixing time tm of 100 ms and a 90 pulse set at 7·70 μs. For each spectrum, a total of 128 scans were accumulated into 64 k data points with a spectral width of 12·001 parts per million. The FID were multiplied by an exponential function corresponding to 0·3 Hz line broadening. All spectra were manually phased, baseline corrected and calibrated to the chemical shift of TSP (*δ* 0·00). Spectra were digitised using an in-house MATLAB (version R2014a; The Mathworks Inc.) script. The spectral region containing the water resonance was removed to minimise distortions in the baseline arising from imperfect water saturation. Principal component analysis (PCA) was performed with Pareto scaling using scripts provided by Korrigan Sciences Ltd.

### Statistical analysis

All statistical tests were performed using GraphPad Prism (version 5.0; Graph-Pad Software). A two-way ANOVA test was used to compare dose, substrates and time-dependent effects. When there was no significant effect, one-way ANOVA tests and paired *t* tests, including *post hoc* tests appropriate for the individual data sets (Bonferroni post-test with significance set at *P*<0·05), were used for bacterial counts and organic acid concentrations.

## Results

### SCFA analysis

**Fig. 1:**
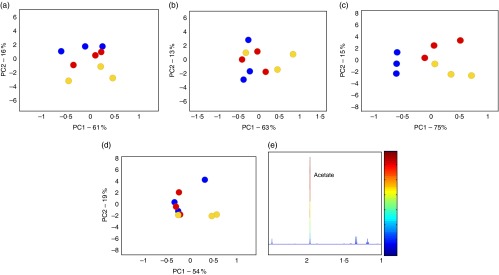
^1^H-NMR data analysis. (a–c) Principal component (PC) analysis (PCA) score plot B-GOS 65 % – T4 and PCA score plot B-GOS 52 % – T4, respectively, show a clear separation during the first 4 h of fermentation due to dose–response effect. (b–d) PCA score plot B-GOS 65 % – T8 and PCA score plot B-GOS 52 % – T8, respectively, show how the dose effect is lost after 4 h of fermentation. (e) The colour plot illustrates the main compound, acetate, influencing the separation. The 65 % B-GOS and 52 % B-GOS were tested at 1, 0·5 and 0·33 g equivalent to 0·1, 0·05 and 0·033 g/l. a, b: 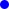, 65 % B-GOS 0·33 g; 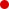, 65 % B-GOS 0·5 g; 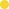, 65 % B-GOS 1 g; c, d: 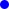, 52 % B-GOS 0·33 g; 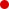, 52 % B-GOS 0·5 g; 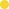, 52 % B-GOS 1 g.

Our results showed acetate as the dominant SCFA produced for both substrates with significant differences between 4 and 8 h of fermentation using 65 % B-GOS at 0·033 g/l (*P*<0·01) (Table 3). In particular, there was a clear dose–response effect during the first 4 h of fermentation. However, no significant differences were observed between the two substrates at the same dose. The dose–response effect was also confirmed by ^1^H-NMR data. PCA revealed a clear trajectory over time, showing a clear separation between 0 and 4 h with acetate as main component influencing variation through time (Fig. 1). Significant decreases in propionate concentration throughout fermentation were observed, mainly between 8 and 24 h of fermentation (*P*<0·01). None of the substrates generated major changes in butyrate production (Table 3). Two-way ANOVA data analyses did not show significant differences on SCFA production between the two substrates.

**Table 3:** SCFA production by GC in the pH-controlled and volume-controlled batch cultures at 0, 4, 8 and 24 h of fermentation† (Mean of the data of three experiments and standard deviations)

		Acetate (mm)		Propionate (mm)		Butyrate (mm)		Branched (mm)		Total (mm)	
Substrates	Time (h)	Mean	sd	Mean	sd	Mean	sd	Mean	sd	Mean	sd
	0	26·77	22·13	6·50	7·25	4·63	5·11	15·60	17·26	53·49	51·53
52 % B-GOS (1 g)	4	30·96	18·64	6·43	8·32	4·44	6·92	16·08	11·36	57·92	56·63
	8	28·39	6·91	2·59	0·76	0·95	0·47	3·13	1·42	35·06	6·89
	24	17·14	0·1	1·06	0·1	0·55	0·1	0·22	0·1	18·98	0·1
	0	17·27	8·37	3·63	3·42	2·57	2·61	8·81	9·28	32·28	23·49
52 % B-GOS (0·5 g)	4	28·33	19·27	4·67	6·09	2·29	3·41	7·71	11·36	43·01	40·13
	8	31·25	15·30	2·11	1·65	0·80	0·51	1·37	0·98	35·52	18·34
	24	20·3	0·1	0·91	0·1	0·39	0·1	0·23	0·1	21·84	0·1
	0	11·41	8·94	2·26	1·92	1·63	1·52	5·74	5·89	21·04	16·22
52 % B-GOS (0·33 g)	4	17·43	19·16	4·56	6·62	1·75	2·63	4·62	7·01	28·36	35·33
	8	26·54	26·11	4·47	5·63	1·24	1·57	1·13	0·92	33·37	35·22
	24	26·24	0·1	0·75	0·1	0·24	0·1	0·17	0·1	27·39	0·1
	0	18·29	10·59	2·33	2·31	1·32	1·39	4·69	4·87	26·63	19·12
65 % B-GOS (1 g)	4	38·42	22·17	4·65	4·45	1·42	1·91	4·47	5·65	48·97	34·11
	8	43·37	28·55	6·81	5·19	1·21	0·99	1·47	1·15	52·82	33·81
	24	6·78	0·1	0·73**	0·1	0·4	0·1	0·39	0·1	8·31	0·1
	0	17·94	7·95	2·01	1·94	0·92	0·97	3·42	3·61	24·29	14·33
65 % B-GOS (0·5 g)	4	30·61	20·92	3·97	3·69	1·27	1·59	3·19	3·95	39·03	30·10
	8	24·43	11·06	3·32	2·47	0·72	0·56	1·18	0·81	29·65	13·96
	24	21·51	0·1	0·61**	0·1	0·50	0·1	0·28	0·1	22·89	0·1
	0	12·29	5·37	1·33	1·14	0·58	0·6	2·1	2·31	16·3	9·21
65 % B-GOS (0·33 g)	4	24·68	15·65	3·39	3·19	0·84	1·03	2·14	2·65	31·05	22·49
	8	27·18**	12·41	3·98	2·72	0·71	0·48	1·25	1·02	33·12	15·48
	24	20·88	0·1	1·18*	0·1	0·97	0·1	0·77	0·1	23·79	0·1
	0	5·28	5·69	0·71	0·51	0·36	0·32	1·15	1·25	7·5	5·34
Negative control	4	8·68	5·25	1·68	0·97	0·61	0·47	1·3	1·66	12·31	8·28
	8	18·82	8·74	2·71	1·47	0·67	0·12	0·97	0·84	23·17	10·64
	24	18·43	0·1	2·37	0·1	1·47	0·1	0·49	0·1	22·76	0·1

B-GOS, Bimuno^®^ galacto-oligosaccharide. * Significantly different from 0 h with *P*<0·05. ** Significantly different from 0 h with *P*<0·01 (one-way ANOVA).

† 52 % B-GOS and 65 % B-GOS at doses 1, 0·5 and 0·33 g (equivalent to 0·1, 0·05 and 0·033 g/l).

### Changes in bacterial populations

A significant increase in bifidobacteria was observed with 52 % B-GOS 0·5 and 0·33 g (equivalent to 0·05 and 0·033 g/l) between time 0 and 24 h and with 65 % B-GOS (*P*<0·05) at same doses at all time points tested (*P*<0·05) (Table 4). Lactobacilli significantly increased after addition of 52 % B-GOS and 65 % B-GOS 1 and 0·33 g (equivalent to 0·1 and 0·033 g/l) (*P*<0·01) and 52 % B-GOS 0·5 g (equivalent to 0·05 g/l) (*P*<0·05) between 8 and 24 h. An overall decrease in the *Bacteroides–Provotella* group with 52 % B-GOS at doses of 1 and 0·33 g (equivalent to 0·1 and 0·033 g/l) was observed between 0 and 24 h (*P*<0·05). With 65 % B-GOS at 1 and 0·5 g (equivalent to 0·1 and 0·05 g/l), there was a significant decrease between 4 and 24 and 0 and 4 h, respectively (*P*<0·05). Two-way ANOVA data analyses did not show significant differences in microbiota composition between the two substrates.

**Table 4:** Bacterial groups detected by fluorescence *in situ* hybridisation in the pH-controlled and volume-controlled batch cultures at 0, 4, 8 and 24 h of fermentation* (Mean of the data of three experiments and standard deviations; *n* 3)

		Bacterial population (log10 cells/ml)													
		52 % B-GOS 1 g		52 % B-GOS 0·5 g		52 % B-GOS 0·33 g		65 % B-GOS 1 g		65% B-GOS 0·5 g		65 % B-GOS 0·33 g		NC	
Probes	Time point (h)	Mean	sd	Mean	sd	Mean	sd	Mean	sd	Mean	sd	Mean	sd	Mean	sd
Eub338 I-II-III	0	9·24	0·09	9·13	0·11	9·22	0·11	9·24	0·11	9·27	0·12	9·23	0·1	9·19	0·08
	4	9·11	0·17	9·15	0·18	9·14	0·44	9·39	0·14	8·9	0·14	9·35	0·07	9·04	0·31
	8	9·16	0·13	9·49	0·23	9·05	0·41	9·52	0·1	8·92	0·1	9·46	0·04	9·2	0·59
	24	9·14	0·01	9·33	0·03	9·1	0·05	8·94^a,*c*^	0·07	9·21	0·01	9·56^a^	0·04	8·95^a^	0·02
Lab158	0	7·63	0·09	7·48	0·11	7·55	0·05	7·52	0·07	7·54	0·15	7·47	0·06	7·48	0·06
	4	7·57	0·05	7·64	0·16	7·77	0·27	7·78	0·14	7·73	0·12	7·31	0·29	7·54	0·2
	8	7·74	0·1	8·05	0·11	7·6	0·36	7·98	0·27	8·21	0·56	7·66	0·12	7·55	0·4
	24	8·39^*a*,**b**,*c*^	0·02	7·70^c^	0·01	7·94^*a*^	0·07	7·94^*a*^	0·09	7·68	0·06	7·85^*a*^	0·05	7·29^a^	0·08
Bif164	0	8·61	0·28	8·7	0·17	8·58	0·26	8·45	0·35	8·63	0·21	8·47	0·3	8·68	0·21
	4	8·73	0·33	9·1	0·22	8·79	0·67	8·86^b^	0·2	8·75	0·19	8·47	0·4	8·45	0·31
	8	8·93	0·29	8·87	0·61	8·95	0·63	9·12	0·21	9·25^a,b^	0·04	9·31^a,b^	0·1	8·53	0·13
	24	9·2	0·06	9·31^a^	0·03	9·19^a^	0·08	9·12	0·21	9·2^a,b^	0·07	9·24^a^	0·04	8·25	0·07
Bac303	0	8·26	0·16	8·23	0·35	8·27	0·33	8·33	0·25	8·33	0·3	8·2	0·19	8·26	0·29
	4	8·07	0·55	8·41	0·53	8·09	0·86	8·49	0·15	7·94^a^	0·26	8·39	0·12	7·75	0·83
	8	7·33	0·64	7·83	0·82	8·11	0·66	8·31	0·46	7·88	0·32	8·63	0·19	6·71^a^	0·37
	24	7·78^a^	0·05	8·39	0·15	7·06^a^	0·2	7·94^b^	0·03	8·41	0·06	8·29	0·04	8·39^a,c^	0·2
Erec482	0	8·35	0·16	8·34	0·13	8·42	0·03	8·34	0·06	8·38	0·09	8·3	0·04	8·32	0·05
	4	8·29	0·12	8·39	0·15	8·17	0·31	8·4	0·21	8·36	0·2	8·24	0·34	8·08	0·43
	8	7·85	1·21	7·92	1·42	7·88	1·22	7·91	1·36	8·45	0·08	8·38	0·38	7·98	0·76
	24	8·85^a,b^	0·37	8·65^a^	0·06	7·94^**a**^	0·04	8·58^a^	0·01	8·59^a^	0·01	8·56^*a*^	0·04	8·06^*a*^	0·05
Rrec584	0	8·34	0·25	8·24	0·08	8·06	0·54	8·15	0·3	8·17	0·17	8·28	0·09	8·34	0·04
	4	7·54	1·04	8·13	0·3	7·85	0·59	8·2	0·41	7·29	0·86	7·77	0·62	7·78	0·69
	8	7·06	0·87	7·47	0·74	7·37^a^	0·78	7·71	0·98	7·21	0·78	7·6	0·56	7·66^a^	0·21
	24	7·2	0·02	7·14^**a**,b^	0·05	6·92	0·01	7·78	0·14	7·23^*a*^	0·06	8·51^a^	0·03	7·54^**a**^	0·2
Chis150	0	5·83	0·2	5·73	1·1	5·83	0·2	5·73	1·1	5·93	0·3	5·73	1·1	5·73	1·1
	4	5·86	0·04	6·34	0·5	5·79	0·1	6·56	0·6	6·11	0·2	6·2	0·3	5·9	0·1
	8	6·03	0·1	6·74	1	6·53	1	6·80	1·1	6·92	1·3	7·24	0·8	5·87	0·2
	24	5·73	0·02	5·73	0·05	7·45	0·01	5·73	0·2	6·43	0·03	5·73	0·01	5·73	0·01
Prop853	0	7·98	0·18	7·97	0·27	8·05	0·22	8·18	0·26	8·16	0·22	8·08	0·22	8·04	0·06
	4	8·04	0·28	7·97	0·02	7·81	0·47	8·02	0·09	8·02	0·05	8·11	0·09	7·99	0·19
	8	7·84	0·37	7·74	0·48	7·83	0·37	7·85^*a*^	0·68	7·67^*a*^	1·03	8·16^a^	0·3	7·76^a^	0·08
	24	8·19^b,c^	0·01	7·99	0·01	7·95^*a*,**b**^	0·04	6·53^**b**^	0·02	6·15^**b**^	0·06	8·72^*b*,c^	0·01	8·1^c^	0·07

B-GOS, Bimuno^®^ galacto-oligosaccharide. ^a^ Significantly different from 0 h with *P*<0·05; ^b^ significantly different from 4 h with *P*<0·05; ^c^ significantly different from 8 h with *P*<0·05; italicised superscript letters were significantly different with *P*<0·01; bold superscript letters were significantly different with *P*<0·001 (*t* test).

* The 65 % B-GOS and 52 % B-GOS were tested at 1, 0·5 and 0·33 g (equivalent to 0·1, 0·05 and 0·033 g/l).

## Discussion

The present study was carried out in order to evaluate the *in vitro* fermentation properties of B-GOS (65 % GOS content) in pH- and volume-controlled batch culture fermentation. The fermentability and the selectivity of GOS have been previously evaluated *in vitro* by several comparative studies. Rycroft *et al*.^(^ ^15^ ^)^ compared the efficacy of different prebiotics including FOS and GOS in 24-h batch culture experiments, and the results show how GOS induced the largest significant increases in bifidobacteria, lactobacilli and total bacterial numbers during fermentation^(^ ^15^ ^)^. In our study, the administration of B-GOS showed the same trend, especially considering the bifidobacteria and lactobacilli population. There was also a significant decrease in *Bacteroidetes* numbers, except for 52 % B-GOS at 0·05 g/l and 65 % B-GOS at 0·033 g/l. The 65 % B-GOS also has a strong influence in the production of SCFA, compared with B-GOS that is commercially available (52 % GOS content). Our results showed a double increase in acetate production at all doses using 65 % B-GOS, but were not significant, except for 65 % B-GOS at 0·033 g/l, probably due to the high standard deviation.

The fermentation of all different doses induced the production of acetate, which correlated with an increase in *Bifidobacterium* populations^(^ ^24^ ^)^. Palframan *et al.* ^(^ ^25^ ^)^ in a study comparing the effect of the pH and dose on batch culture fermentation of five commercial prebiotics have shown similar results. FISH analysis showed how the highest bacterial numbers were obtained with GOS at pH 6 and 1 % (w/v)^(^ ^25^ ^)^. The global effect on the bacterial population of 52 % B-GOS has been tested in a previous fermentation study where the commercial B-GOS mixture was compared with different purified GOS. Using the Selectivity Index (SI) as a value for the growth of beneficial bacteria, Rodriguez-Colinas *et al*.^(^ ^26^ ^)^ have shown that 52 % B-GOS had the highest SI, and consequently a strong degree of selectivity for *Bifidobacterium* population.

Our results highlighted that 65 % GOS had an effective prebiotic activity, in terms of increasing the number of bifidobacteria and metabolite production. This was especially seen for acetate, probably due to the lower content of monosaccharides and disaccharides in the mixture that might have affected *in vitro* fermentation experiments overall. However, a previous study of Costabile *et al*.^(^ ^27^ ^)^ has demonstrated that the carbohydrates that remained after the *in vitro* pre-digestion process did not have any selective properties to invoke a bifidogenic effect, which perhaps would not persist *in vivo* ^(^ ^28^ ^)^.

In our study, major changes in other bacterial populations were seen, which might be due to the presence of these sugars. Different concentrations of SCFA have been identified at time 0, but it may be explained by the high inter-individual variability among each individual. Significant differences were observed between the time points in all analyses but not between the two substrates.

The effects of B-GOS 52 % as a potential modulator of the gut microflora and the immune system have been extensively investigated in several human intervention studies. The 65 % B-GOS has shown a significant modulation of health-promoting beneficial bacteria, and our findings proved that reducing impurities in the prebiotic mixture might improve the selectivity of prebiotics in *in vitro* experiments. However, the comparison between the effect of 65 % B-GOS and 52 % B-GOS has shown similar bifidogenic effects (data not published). The applicability of such changes remains to be investigated in *in vivo* human intervention studies.
